# Methyl 7,8-diacet­oxy-11-oxo-5-(2-oxo­pyrrolidin-1-yl)-7,9-epoxy­cyclo­penta­[4,5]pyrido[1,2-*a*]quinoline-10-carboxyl­ate sesquihydrate

**DOI:** 10.1107/S160053680905363X

**Published:** 2009-12-19

**Authors:** Atash V. Gurbanov, Eugeniya V. Nikitina, Vladimir P. Zaytsev, Fedor I. Zubkov, Victor N. Khrustalev

**Affiliations:** a Baku State University, Z. Khalilov St 23, Baku AZ-1148, Azerbaijan; b Organic Chemistry Department, Russian Peoples Friendship University, Miklukho-Maklaya St 6, Moscow 117198, Russian Federation; c X-Ray Structural Centre, A. N.Nesmeyanov Institute of Organoelement Compounds, Russian Academy of Sciences, 28 Vavilov St, B-334, Moscow 119991, Russian Federation

## Abstract

The title compound, C_26_H_28_N_2_O_9_·1.5H_2_O, the product of an acid-catalysed Wagner–Meerwein skeletal rearrangement, crystallizes as a sesquihydrate with the O atom of one of the two independent water mol­ecules occupying a special position on a twofold axis. The organic mol­ecule comprises a fused penta­cyclic system containing two five-membered rings (cyclo­pentane and tetra­hydro­furan) and three six-membered rings (piperidinone, tetra­hydro­pyridine and benzene). The five-membered rings have the usual envelope conformations, and the central six-membered piperidinone and tetra­hydro­pyridine rings adopt boat and sofa conformations, respectively. In the crystal, there are three independent O—H⋯O hydrogen bonds, which link the organic mol­ecules and water mol­ecules into complex two-tier layers parallel to (001). The layers are further linked into a three-dimensional framework by attractive inter­molecular carbon­yl–carbonyl inter­actions.

## Related literature

For general background to the use of acid-catalysed Wagner-Meerwein rearrangement of substituted 3,8-dioxatricyclo­[3.2.1.0^2,4^]octa­nes (ep­oxy-7-oxabicyclo­[2.2.1]heptenes) in organic synthesis, see: Popp & McEwen (1958 ▶); Hogeveen & Van Krutchten (1979 ▶); Hanson (1991 ▶). For related structures, see: Jung & Street (1985 ▶); Keay *et al.* (1989 ▶); Zubkov *et al.* (2004 ▶, 2007 ▶, 2009 ▶); Gurbanov *et al.* (2009 ▶). For carbon­yl–carbonyl inter­actions, see: Allen *et al.* (1998 ▶).
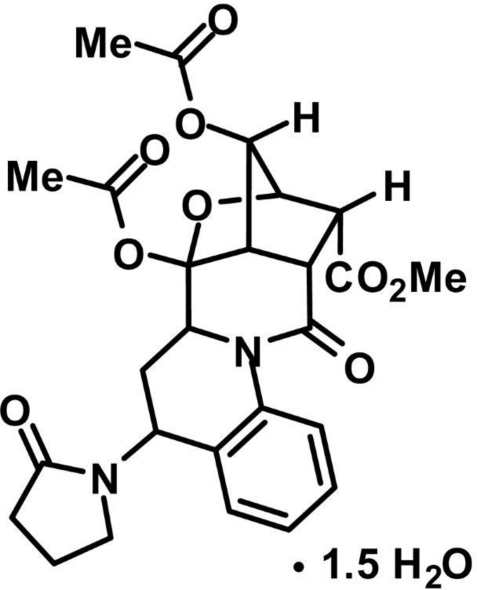


## Experimental

### 

#### Crystal data

C_26_H_28_N_2_O_9_·1.5H_2_O
*M* *_r_* = 539.53
Monoclinic, 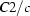

*a* = 16.8557 (5) Å
*b* = 9.9692 (3) Å
*c* = 29.6704 (8) Å
β = 90.035 (1)°
*V* = 4985.7 (2) Å^3^
*Z* = 8
Mo *K*α radiation
μ = 0.11 mm^−1^
*T* = 100 K
0.30 × 0.20 × 0.20 mm

#### Data collection

Bruker SMART APEXII CCD diffractometer
Absorption correction: multi-scan (*SADABS*; Sheldrick, 2003 ▶) *T*_min_ = 0.969, *T*_max_ = 0.979
29541 measured reflections
6460 independent reflections
5531 reflections with *I* > 2σ(*I*)
*R*_int_ = 0.031

#### Refinement

*R*[*F* ^2^ > 2σ(*F* ^2^)] = 0.040
*wR*(*F* ^2^) = 0.106
*S* = 1.00
6460 reflections
351 parameters
H-atom parameters constrained
Δρ_max_ = 0.45 e Å^−3^
Δρ_min_ = −0.27 e Å^−3^

### 

Data collection: *APEX2* (Bruker, 2005 ▶); cell refinement: *SAINT-Plus* (Bruker, 2001 ▶); data reduction: *SAINT-Plus*; program(s) used to solve structure: *SHELXTL* (Sheldrick, 2008 ▶); program(s) used to refine structure: *SHELXTL*; molecular graphics: *SHELXTL*; software used to prepare material for publication: *SHELXTL*.

## Supplementary Material

Crystal structure: contains datablocks global, I. DOI: 10.1107/S160053680905363X/ya2114sup1.cif

Structure factors: contains datablocks I. DOI: 10.1107/S160053680905363X/ya2114Isup2.hkl

Additional supplementary materials:  crystallographic information; 3D view; checkCIF report

## Figures and Tables

**Table 1:** Hydrogen-bond geometry (Å, °)

*D*—H⋯*A*	*D*—H	H⋯*A*	*D*⋯*A*	*D*—H⋯*A*
O9—H9*B*⋯O3^i^	0.89	1.98	2.8576 (12)	173
O9—H9*C*⋯O8	0.86	1.98	2.8365 (13)	177
O10—H10*C*⋯O1	0.91	2.06	2.9516 (15)	167

Symmetry code: (i) 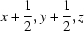.

**Fig. 1.**
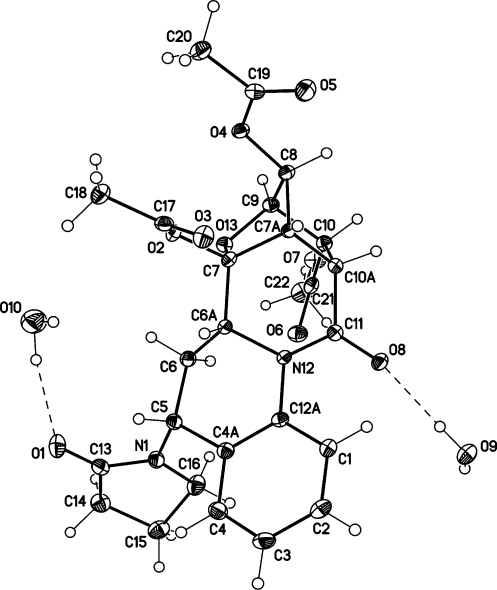
Molecular structure of the title compound. Displacement ellipsoids are drawn at the 50% probability level. Dashed lines show intermolecular hydrogen bonds.

**Fig. 2.**
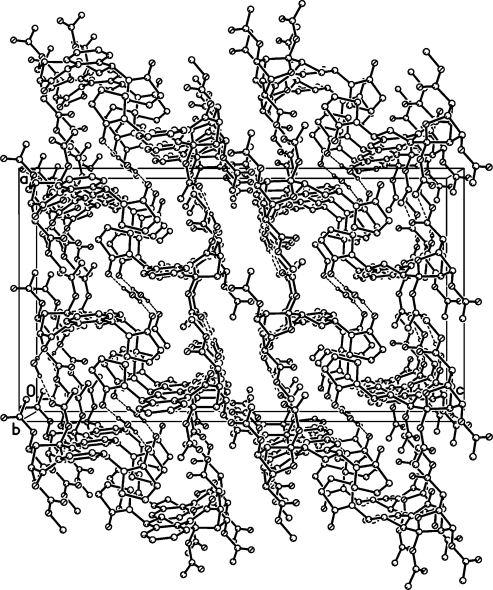
Crystal packing of the title compound viewed down the b axis. Dashed lines show intermolecular hydrogen bonds.

**Fig. 3.**
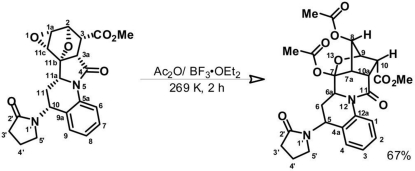
Wagner-Meerwein skeletal rearrangement of methyl 4-oxo-10-(2-oxopyrrolidinyl)-10H-2,11b-epoxyoxireno[6,7]isoindolo[2,1-a]quinoline-3-carboxylate.

## supplementary crystallographic information

### Comment

Acid catalyzed Wagner-Meerwein rearrangement of substituted 3,8-dioxatricyclo[3.2.1.0^2,4^]octanes (epoxy-7-oxabicyclo[2.2.1]heptenes) is used extensively in organic synthesis (Popp & McEwen,1958; Hogeveen & Van Krutchten, 1979; Hanson, 1991). However, with the exception of our works (Zubkov *et al.* 2004; Zubkov *et al.* 2009; Gurbanov *et al. *2009), only a few examples of the skeletal rearrangement for 7-oxabicyclo[2.2.1]heptenes condensed with nitrogen-rings are known to date (Jung & Street, 1985; Keay *et al.* 1989). In particular, Wagner-Meerwein rearrangement in isoindolo[2,1-*a*]quinolines series has not been studied yet. The present work is meant to cover this gap. Hereunder, we report a new diastereoselective approach to 7,8-bis(acetyloxy)-5-*R*-7,9-epoxycyclopenta[4,5]pyrido[1,2-*a*]quinolines using 2-furyltetrahydroquinolines, readily accessible by Povarov reaction, as starting compounds (Zubkov *et al.* (2007)).

The structure of methyl 7,8-bis(acetyloxy)-11-oxo-5-(*N*-pyrrolidonyl)-7,9-epoxycyclopenta[4,5]pyrido[1,2-*a*]quinoline-10-carboxylate (I) was established by X-ray diffraction study (Fig. 1). Compound (I) crystallizes as sesquihydrate, *i. e.*, C_26_H_28_N_2_O_9_.1.5H_2_O; one of the two independent water molecules occupies a special position on the twofold axis. Molecule of (I) comprises a fused pentacyclic system containing two five-membered rings (cyclopentane and tetrahydrofuran) and three six-membered rings (piperidinone, tetrahydropyridine and benzene). Both five-membered rings of the bicyclic fragment have usual *envelope *conformations, and the central six-membered piperidinone and tetrahydropyridine rings adopt the *boat* and *sofa* conformations, respectively. The nitrogen N12 atom has a trigonal-planar geometry (sum of the bond angles is 359.5°). The dihedral angle between the planes of the piperidinone (or rather "bottom of the boat" C6A, C7, C10A, C11 plane) and benzene is 38.81 (3)°. The two carboxylate substituents at the C7 and C8 carbon atoms are in the sterically unfavorable *syn*-periplanar configuration relative to the tetrahydrofuran ring. Such disposition is explained by the direction of the Wagner-Meerwein rearrangement.

The pyrrolidinone ligand and carboxylate substituent at the C10 atom are on the same side of the core pentacyclic framework. It is noteworthy that these fragments are engaged in the attractive intermolecular carbonyl-carbonyl interactions C13═O1···C17^i^═ O3^i^ and C19═O5···C11^ii^═ O8^ii^ [O1···C17^i^ 2.947 (1) Å, O5···C11^ii^ 2.993 (1) Å; symmetry codes: (i) -*x*, *y*, 1/2 - *z*; (ii) -*x*, 1 - *y*, 1 - *z*]. Carbonyl-carbonyl interactions of such type were investigated in substantial detail by Allen *et al.* (1998).

The molecule of (I) possesses eight asymmetric centers at the C5, C6A, C7, C7A, C8, C9, C10 and C10A atoms and can have potentially numerous diastereomers. The crystal of (I) is racemic and consists of enantiomeric pairs with the following relative configuration of the centers: *rac*-5*S**,6a*S**,7*R**,7a*R**,8*R**,9*S**,10*S**,10*aR**.

There are three independent H-bonds (Table 1), which link the molecules of (I) and water molecules into complex two-tier layers parallel to (001) (Fig. 2). The layers are linked further into three-dimensional framework by the above-mentioned attractive intermolecular carbonyl-carbonyl interactions.

### Experimental

Boron trifluoride etherate (0.3 ml, 2.4 mmol) was added to a solution of methyl (1a*R**,2*R**,3*R**,3a*S**,10*R**,11*aR**,11*bR**,11*cR**)-4-oxo-10-(2-oxopyrrolidin-1-yl)-1a,2,3,3a,4,11,11*a*,11*c*-octahydro-10*H*-2,11*b*-epoxyoxireno[6,7]isoindolo[2,1-*a*]quinoline-3-carboxylate (0.51 g, 1.2 mmol) in 15 ml of acetic anhydride. The mixture was stirred for 2 h at 296 K, then it was diluted with 100 ml of water and treated with a saturated solution of sodium carbonate until pH ~ 8–9. Organic products were extracted with chloroform (3 *x* 50 ml). The extract was dried (MgSO_4_), concentrated and purified by crystallization from hexane-ethyl acetate to give 0.41 g (0.8 mmol) of the polycycle (I) as colourless rhomboid-shaped blocks (Fig. 3). Yield is 67%. The single crystals of product (I) were obtained by slow crystallization from a mixture of 95% ethanol-DMF (yield 30%). *M*.p. = 460–462 K. IR, ν (cm^-1^): 1673, 1744 (NCO, CO_2_*Me*, CO*Me*). Mass spectrum, m/*z* (I_r_(%)): 512 [*M*^+^] (3), 452 (100), 397 (38), 363 (32), 308 (31), 280 (55), 265 (28), 248 (22), 213 (47), 196 (25), 130 (86), 43 (62). ^1^H NMR (CDCl_3_, 293 K): δ = 7.87 (dd, 1H, H1, J_1,2_ = 8.4, J_1,3_ = 1.2), 7.18 (ddd, 1H, H2, J_1,2_ = 8.4, J_2,3_ = 6.9, J_2,4_ = 2.0), 7.06 (ddd, 1H, H3, J_3,4_ = 7.9, J_2,3_ = 6.9, J_1,3_ = 1.2), 7.02 (d, 1H, H4, J_3,4_ = 7.9, J_2,4_ = 2.0), 5.56 (dd, 1H, H5, J_5,6 A_ = 11.3, J_5,6B_ = 8.2), 4.93 (d, 1H, H8, J_7a,8_ = 1.9), 4.76 (bs, 1H, H9), 4.22 (dd, 1H, H6a, J_6a,6 A_ = 12.0, J_6a,6B_ = 1.7), 4.05 (dd, 1H, H7a, J_7a,10_*_a_* = 4.7, J_7a,8_ = 1.9), 3.59 (s, 3H, CO2*Me*), 3.36 (dd, 1H, H10*a*, J_7a,10_*_a_* = 4.7, J_10,10_*_a_* = 11.3), 3.33 (m, 1H, H5A'), 3.23 (d, 1H, H10, J_10,10_*_a_* = 11.3), 3.00 (m, 1H, H5B'), 2.54 (ddd, 1H, H6A, J_6 A,6B_ = 13.3, J_6a,6 A_ = 12.0, J_5,6 A_ = 11.3), 2.43 (m, 2H, H3'), 2.28 (ddd, 1H, H6B, J_6 A,6B_ = 13.3, J_5,6B_ = 8.2, J_6a,6B_ = 1.7), 2.07 (s, 3H, CO*Me*), 2.05 (s, 3H, CO*Me*), 1.93 (m, 2H, H4'). ^13^C NMR (CDC1_3_, 293 K): δ = 175.5 (s, C2'), 169.1, 169.7 (s, CO*Me*), 168.3 (s, CO_2_*Me*), 165.0 (s, C11), 137.9 (s, C12*a*), 127.6 (d, C4, J = 158.6), 127.3 (d, C2, J = 161.4), 126.4 (s, C4a), 125.5 (d, C3, J = 162.0), 125.1 (d, C1, J = 165.7), 106.2 (s, C7), 81.4 (d, C9, J = 172.0), 76.3 (d, C8, J = 159.0), 58.5 (d, C6a, J = 142.7), 52.1 (q, CO_2_*Me*, J = 147.7), 47.6 (d, C5, J = 139.0), 46.4 (d, C10, J = 139.0), 42.0 (t, C5', J = 145.0), 41.3 (d, C7a, J = 158.5), 39.3 (d, C10*a*, J = 143.5), 31.1 (t, C3', J = 133.0), 25.2 (t, C6, J = 134.0), 20.6, 21.7 (q, CO*Me*, J = 130.2), 17.8 (t, C4', J = 134.0).

### Refinement

The hydrogen atoms of the solvate water molecules were located in the difference Fourier map; C-bound H atoms were placed geometrically (C—H 0.95 Å, 0.98 Å, 0.99 Å, and 1.00 Å for aromatic, methyl, methylene and methine H atoms respectively) and included in the refinement in riding motion approximation [*U*_iso_(H) = 1.2*U*_eq_(C) for non-methyl H atoms; *U*_iso_(H) = 1.5*U*_eq_ of the caryying atom for methyl and O-bound H atoms].

### Crystal data

**Table:**

C_26_H_28_N_2_O_9_·1.5H_2_O	*F*(000) = 2280
*M**_r_* = 539.53	*D*_x_ = 1.438 Mg m^−^^3^
Monoclinic, *C*2/*c*	Mo *K*α radiation, λ = 0.71073 Å
Hall symbol: -C 2yc	Cell parameters from 9578 reflections
*a* = 16.8557 (5) Å	θ = 2.4–28.6°
*b* = 9.9692 (3) Å	µ = 0.11 mm^−^^1^
*c* = 29.6704 (8) Å	*T* = 100 K
β = 90.035 (1)°	Prism, colourless
*V* = 4985.7 (2) Å^3^	0.30 × 0.20 × 0.20 mm
*Z* = 8	

### Data collection

**Table:**

Bruker SMART APEXII CCD diffractometer	6460 independent reflections
Radiation source: fine-focus sealed tube	5531 reflections with *I* > 2σ(*I*)
graphite	*R*_int_ = 0.031
φ and ω scans	θ_max_ = 28.7°, θ_min_ = 1.4°
Absorption correction: multi-scan (*SADABS*; Sheldrick, 2003)	*h* = −22→22
*T*_min_ = 0.969, *T*_max_ = 0.979	*k* = −13→13
29541 measured reflections	*l* = −40→40

### Refinement

**Table:**

Refinement on *F*^2^	Primary atom site location: structure-invariant direct methods
Least-squares matrix: full	Secondary atom site location: difference Fourier map
*R*[*F*^2^ > 2σ(*F*^2^)] = 0.040	Hydrogen site location: difference Fourier map
*wR*(*F*^2^) = 0.106	H-atom parameters constrained
*S* = 1.00	*w* = 1/[σ^2^(*F*_o_^2^) + (0.054*P*)^2^ + 5.5*P*] where *P* = (*F*_o_^2^ + 2*F*_c_^2^)/3
6460 reflections	(Δ/σ)_max_ = 0.001
351 parameters	Δρ_max_ = 0.45 e Å^−^^3^
0 restraints	Δρ_min_ = −0.27 e Å^−^^3^

### Special details

**Table:**

Geometry. All e.s.d.'s (except the e.s.d. in the dihedral angle between two l.s. planes) are estimated using the full covariance matrix. The cell e.s.d.'s are taken into account individually in the estimation of e.s.d.'s in distances, angles and torsion angles; correlations between e.s.d.'s in cell parameters are only used when they are defined by crystal symmetry. An approximate (isotropic) treatment of cell e.s.d.'s is used for estimating e.s.d.'s involving l.s. planes.
Refinement. Refinement of *F*^2^ against ALL reflections. The weighted *R*-factor *wR* and goodness of fit *S* are based on *F*^2^, conventional *R*-factors *R* are based on *F*, with *F* set to zero for negative *F*^2^. The threshold expression of *F*^2^ > σ(*F*^2^) is used only for calculating *R*-factors(gt) *etc*. and is not relevant to the choice of reflections for refinement. *R*-factors based on *F*^2^ are statistically about twice as large as those based on *F*, and *R*- factors based on ALL data will be even larger.

### Fractional atomic coordinates and isotropic or equivalent isotropic displacement parameters (Å^2^)

**Table:**

	*x*	*y*	*z*	*U*_iso_*/*U*_eq_	
O1	0.08324 (6)	0.49746 (10)	0.20077 (3)	0.0214 (2)	
O2	0.00452 (5)	0.32130 (8)	0.37846 (3)	0.01336 (17)	
O3	−0.08727 (5)	0.45915 (10)	0.40862 (3)	0.01900 (19)	
O4	0.03384 (5)	0.19384 (8)	0.45989 (3)	0.01436 (18)	
O5	−0.02640 (6)	0.27537 (10)	0.52181 (3)	0.0218 (2)	
O6	0.28555 (5)	0.48346 (9)	0.38299 (3)	0.01877 (19)	
O7	0.33899 (5)	0.31262 (10)	0.42233 (3)	0.0201 (2)	
O8	0.20831 (5)	0.72008 (9)	0.43497 (3)	0.01602 (18)	
O13	0.13523 (5)	0.30526 (8)	0.38994 (3)	0.01262 (17)	
N1	0.15989 (6)	0.61656 (11)	0.25045 (3)	0.0150 (2)	
N12	0.11703 (6)	0.63699 (10)	0.38547 (3)	0.01132 (19)	
C1	0.10122 (7)	0.88174 (12)	0.37973 (4)	0.0144 (2)	
H1A	0.1086	0.8900	0.4114	0.017*	
C2	0.08267 (7)	0.99401 (13)	0.35419 (5)	0.0178 (2)	
H2A	0.0771	1.0788	0.3684	0.021*	
C3	0.07217 (8)	0.98311 (13)	0.30770 (5)	0.0193 (3)	
H3A	0.0585	1.0596	0.2903	0.023*	
C4	0.08197 (7)	0.85918 (13)	0.28724 (4)	0.0173 (2)	
H4A	0.0762	0.8521	0.2555	0.021*	
C4A	0.10008 (7)	0.74439 (12)	0.31227 (4)	0.0135 (2)	
C5	0.10420 (7)	0.60998 (12)	0.28818 (4)	0.0133 (2)	
H5A	0.0505	0.5934	0.2750	0.016*	
C6	0.12172 (7)	0.49241 (12)	0.31990 (4)	0.0137 (2)	
H6A	0.1026	0.4074	0.3065	0.016*	
H6B	0.1795	0.4847	0.3252	0.016*	
C6A	0.07874 (7)	0.52004 (11)	0.36417 (4)	0.0113 (2)	
H6C	0.0234	0.5476	0.3563	0.014*	
C7	0.07219 (7)	0.39834 (11)	0.39507 (4)	0.0114 (2)	
C7A	0.07365 (7)	0.43157 (11)	0.44532 (4)	0.0116 (2)	
H7A	0.0254	0.4772	0.4576	0.014*	
C8	0.09388 (7)	0.29490 (12)	0.46591 (4)	0.0131 (2)	
H8A	0.1106	0.3029	0.4981	0.016*	
C9	0.16441 (7)	0.27200 (12)	0.43475 (4)	0.0131 (2)	
H9A	0.1901	0.1819	0.4374	0.016*	
C10	0.21730 (7)	0.39149 (12)	0.44830 (4)	0.0132 (2)	
H10A	0.2409	0.3741	0.4787	0.016*	
C10A	0.15354 (7)	0.50606 (12)	0.45219 (4)	0.0119 (2)	
H10B	0.1544	0.5376	0.4842	0.014*	
C11	0.16372 (7)	0.62952 (12)	0.42287 (4)	0.0124 (2)	
C12A	0.10911 (7)	0.75633 (12)	0.35899 (4)	0.0123 (2)	
C13	0.14485 (7)	0.55735 (13)	0.21039 (4)	0.0155 (2)	
C14	0.21733 (8)	0.57640 (14)	0.18066 (4)	0.0197 (3)	
H14A	0.2018	0.6014	0.1496	0.024*	
H14B	0.2500	0.4939	0.1797	0.024*	
C15	0.26181 (8)	0.69058 (14)	0.20379 (5)	0.0216 (3)	
H15A	0.2436	0.7790	0.1926	0.026*	
H15B	0.3197	0.6828	0.1990	0.026*	
C16	0.24042 (8)	0.67116 (14)	0.25359 (5)	0.0202 (3)	
H16A	0.2769	0.6073	0.2685	0.024*	
H16B	0.2411	0.7574	0.2701	0.024*	
C17	−0.07050 (7)	0.35993 (12)	0.38683 (4)	0.0145 (2)	
C18	−0.12828 (8)	0.26323 (14)	0.36648 (5)	0.0205 (3)	
H18A	−0.1816	0.2819	0.3780	0.031*	
H18B	−0.1131	0.1714	0.3745	0.031*	
H18C	−0.1280	0.2730	0.3336	0.031*	
C19	−0.02611 (7)	0.19880 (12)	0.49049 (4)	0.0150 (2)	
C20	−0.09028 (8)	0.09958 (14)	0.47986 (5)	0.0210 (3)	
H20A	−0.1002	0.0432	0.5063	0.032*	
H20B	−0.0736	0.0431	0.4545	0.032*	
H20C	−0.1390	0.1476	0.4718	0.032*	
C21	0.28295 (7)	0.40599 (12)	0.41401 (4)	0.0144 (2)	
C22	0.40460 (8)	0.30901 (15)	0.39090 (5)	0.0238 (3)	
H22A	0.4416	0.2377	0.3996	0.036*	
H22B	0.4322	0.3955	0.3913	0.036*	
H22C	0.3845	0.2914	0.3605	0.036*	
O9	0.25847 (5)	0.98922 (9)	0.44722 (3)	0.01930 (19)	
H9B	0.3064	0.9874	0.4350	0.029*	
H9C	0.2420	0.9089	0.4431	0.029*	
O10	0.0000	0.28215 (16)	0.2500	0.0342 (4)	
H10C	0.0262	0.3392	0.2313	0.051*	

### Atomic displacement parameters (Å^2^)

**Table:**

	*U*^11^	*U*^22^	*U*^33^	*U*^12^	*U*^13^	*U*^23^
O1	0.0179 (4)	0.0277 (5)	0.0185 (4)	−0.0027 (4)	0.0003 (3)	−0.0069 (4)
O2	0.0130 (4)	0.0114 (4)	0.0157 (4)	−0.0023 (3)	−0.0013 (3)	−0.0015 (3)
O3	0.0147 (4)	0.0209 (5)	0.0214 (5)	0.0001 (3)	0.0021 (3)	−0.0032 (4)
O4	0.0158 (4)	0.0125 (4)	0.0148 (4)	−0.0026 (3)	0.0036 (3)	−0.0001 (3)
O5	0.0213 (5)	0.0226 (5)	0.0214 (5)	−0.0037 (4)	0.0067 (4)	−0.0056 (4)
O6	0.0165 (4)	0.0191 (4)	0.0208 (5)	−0.0011 (3)	0.0021 (3)	0.0033 (4)
O7	0.0137 (4)	0.0207 (5)	0.0260 (5)	0.0043 (3)	0.0035 (4)	0.0039 (4)
O8	0.0160 (4)	0.0141 (4)	0.0180 (4)	−0.0033 (3)	−0.0019 (3)	−0.0005 (3)
O13	0.0133 (4)	0.0111 (4)	0.0134 (4)	0.0024 (3)	0.0011 (3)	−0.0004 (3)
N1	0.0146 (5)	0.0183 (5)	0.0122 (5)	−0.0034 (4)	0.0021 (4)	−0.0009 (4)
N12	0.0135 (5)	0.0090 (4)	0.0115 (4)	−0.0016 (3)	0.0002 (4)	−0.0003 (3)
C1	0.0136 (5)	0.0135 (5)	0.0161 (5)	−0.0005 (4)	0.0003 (4)	−0.0016 (4)
C2	0.0163 (6)	0.0119 (5)	0.0253 (6)	−0.0001 (4)	0.0005 (5)	−0.0003 (5)
C3	0.0183 (6)	0.0145 (6)	0.0251 (7)	0.0001 (5)	−0.0018 (5)	0.0062 (5)
C4	0.0172 (6)	0.0187 (6)	0.0161 (6)	−0.0013 (5)	−0.0015 (5)	0.0031 (5)
C4A	0.0116 (5)	0.0144 (5)	0.0145 (5)	−0.0011 (4)	0.0011 (4)	0.0006 (4)
C5	0.0135 (5)	0.0151 (5)	0.0113 (5)	−0.0015 (4)	0.0013 (4)	0.0000 (4)
C6	0.0166 (5)	0.0120 (5)	0.0125 (5)	−0.0002 (4)	0.0015 (4)	−0.0012 (4)
C6A	0.0128 (5)	0.0100 (5)	0.0112 (5)	−0.0010 (4)	−0.0001 (4)	−0.0005 (4)
C7	0.0101 (5)	0.0104 (5)	0.0138 (5)	−0.0013 (4)	0.0006 (4)	−0.0010 (4)
C7A	0.0124 (5)	0.0104 (5)	0.0119 (5)	−0.0008 (4)	0.0011 (4)	0.0001 (4)
C8	0.0137 (5)	0.0113 (5)	0.0144 (5)	−0.0015 (4)	0.0008 (4)	0.0004 (4)
C9	0.0126 (5)	0.0122 (5)	0.0145 (5)	0.0009 (4)	0.0000 (4)	0.0017 (4)
C10	0.0125 (5)	0.0130 (5)	0.0140 (5)	0.0007 (4)	−0.0005 (4)	0.0008 (4)
C10A	0.0123 (5)	0.0119 (5)	0.0115 (5)	−0.0002 (4)	−0.0003 (4)	0.0002 (4)
C11	0.0116 (5)	0.0128 (5)	0.0127 (5)	0.0008 (4)	0.0019 (4)	−0.0011 (4)
C12A	0.0107 (5)	0.0113 (5)	0.0149 (5)	−0.0004 (4)	0.0008 (4)	0.0016 (4)
C13	0.0176 (6)	0.0154 (6)	0.0135 (5)	0.0022 (4)	0.0011 (4)	0.0009 (4)
C14	0.0200 (6)	0.0225 (6)	0.0167 (6)	0.0002 (5)	0.0058 (5)	−0.0004 (5)
C15	0.0210 (6)	0.0198 (6)	0.0241 (7)	−0.0025 (5)	0.0083 (5)	0.0002 (5)
C16	0.0159 (6)	0.0231 (6)	0.0215 (6)	−0.0054 (5)	0.0032 (5)	−0.0035 (5)
C17	0.0134 (5)	0.0158 (5)	0.0143 (5)	−0.0022 (4)	0.0007 (4)	0.0042 (4)
C18	0.0175 (6)	0.0206 (6)	0.0234 (6)	−0.0071 (5)	−0.0028 (5)	0.0011 (5)
C19	0.0151 (5)	0.0139 (5)	0.0159 (6)	0.0007 (4)	0.0018 (4)	0.0030 (4)
C20	0.0198 (6)	0.0208 (6)	0.0224 (6)	−0.0065 (5)	0.0024 (5)	0.0001 (5)
C21	0.0122 (5)	0.0137 (5)	0.0173 (6)	−0.0017 (4)	−0.0012 (4)	−0.0019 (4)
C22	0.0150 (6)	0.0259 (7)	0.0303 (7)	0.0029 (5)	0.0068 (5)	−0.0008 (6)
O9	0.0175 (4)	0.0153 (4)	0.0252 (5)	−0.0005 (3)	0.0021 (4)	−0.0030 (4)
O10	0.0438 (10)	0.0258 (8)	0.0331 (9)	0.000	0.0011 (7)	0.000

### Geometric parameters (Å, °)

**Table:**

O1—C13	1.2314 (16)	C7—C7A	1.5276 (16)
O2—C17	1.3453 (15)	C7A—C8	1.5316 (16)
O2—C7	1.4605 (13)	C7A—C10A	1.5512 (16)
O3—C17	1.2151 (16)	C7A—H7A	1.0000
O4—C19	1.3597 (15)	C8—C9	1.5237 (16)
O4—C8	1.4391 (14)	C8—H8A	1.0000
O5—C19	1.2027 (16)	C9—C10	1.5410 (16)
O6—C21	1.2023 (15)	C9—H9A	1.0000
O7—C21	1.3488 (15)	C10—C21	1.5107 (16)
O7—C22	1.4475 (16)	C10—C10A	1.5725 (16)
O8—C11	1.2282 (15)	C10—H10A	1.0000
O13—C7	1.4189 (14)	C10A—C11	1.5171 (16)
O13—C9	1.4558 (14)	C10A—H10B	1.0000
N1—C13	1.3509 (16)	C13—C14	1.5192 (17)
N1—C5	1.4629 (15)	C14—C15	1.5258 (19)
N1—C16	1.4652 (16)	C14—H14A	0.9900
N12—C11	1.3620 (15)	C14—H14B	0.9900
N12—C12A	1.4320 (15)	C15—C16	1.5334 (19)
N12—C6A	1.4747 (14)	C15—H15A	0.9900
C1—C2	1.3872 (17)	C15—H15B	0.9900
C1—C12A	1.3999 (16)	C16—H16A	0.9900
C1—H1A	0.9500	C16—H16B	0.9900
C2—C3	1.3948 (19)	C17—C18	1.4972 (17)
C2—H2A	0.9500	C18—H18A	0.9800
C3—C4	1.3865 (18)	C18—H18B	0.9800
C3—H3A	0.9500	C18—H18C	0.9800
C4—C4A	1.3979 (17)	C19—C20	1.4991 (17)
C4—H4A	0.9500	C20—H20A	0.9800
C4A—C12A	1.3994 (17)	C20—H20B	0.9800
C4A—C5	1.5203 (17)	C20—H20C	0.9800
C5—C6	1.5316 (16)	C22—H22A	0.9800
C5—H5A	1.0000	C22—H22B	0.9800
C6—C6A	1.5256 (16)	C22—H22C	0.9800
C6—H6A	0.9900	O9—H9B	0.8858
C6—H6B	0.9900	O9—H9C	0.8567
C6A—C7	1.5248 (16)	O10—H10C	0.9089
C6A—H6C	1.0000		
			
C17—O2—C7	121.40 (9)	C21—C10—C9	108.79 (10)
C19—O4—C8	114.49 (9)	C21—C10—C10A	118.74 (10)
C21—O7—C22	115.75 (10)	C9—C10—C10A	100.68 (9)
C7—O13—C9	107.67 (8)	C21—C10—H10A	109.4
C13—N1—C5	122.24 (10)	C9—C10—H10A	109.4
C13—N1—C16	113.05 (10)	C10A—C10—H10A	109.4
C5—N1—C16	124.25 (10)	C11—C10A—C7A	114.30 (9)
C11—N12—C12A	123.10 (10)	C11—C10A—C10	118.02 (9)
C11—N12—C6A	123.95 (10)	C7A—C10A—C10	103.65 (9)
C12A—N12—C6A	112.39 (9)	C11—C10A—H10B	106.7
C2—C1—C12A	120.12 (11)	C7A—C10A—H10B	106.7
C2—C1—H1A	119.9	C10—C10A—H10B	106.7
C12A—C1—H1A	119.9	O8—C11—N12	123.44 (11)
C1—C2—C3	120.38 (12)	O8—C11—C10A	119.88 (11)
C1—C2—H2A	119.8	N12—C11—C10A	116.49 (10)
C3—C2—H2A	119.8	C4A—C12A—C1	120.08 (11)
C4—C3—C2	119.17 (12)	C4A—C12A—N12	118.88 (10)
C4—C3—H3A	120.4	C1—C12A—N12	120.64 (10)
C2—C3—H3A	120.4	O1—C13—N1	125.01 (12)
C3—C4—C4A	121.52 (12)	O1—C13—C14	127.19 (12)
C3—C4—H4A	119.2	N1—C13—C14	107.80 (11)
C4A—C4—H4A	119.2	C13—C14—C15	103.14 (10)
C4—C4A—C12A	118.70 (11)	C13—C14—H14A	111.1
C4—C4A—C5	118.80 (11)	C15—C14—H14A	111.1
C12A—C4A—C5	122.39 (11)	C13—C14—H14B	111.1
N1—C5—C4A	110.47 (10)	C15—C14—H14B	111.1
N1—C5—C6	112.39 (10)	H14A—C14—H14B	109.1
C4A—C5—C6	113.24 (10)	C14—C15—C16	102.90 (10)
N1—C5—H5A	106.8	C14—C15—H15A	111.2
C4A—C5—H5A	106.8	C16—C15—H15A	111.2
C6—C5—H5A	106.8	C14—C15—H15B	111.2
C6A—C6—C5	107.42 (9)	C16—C15—H15B	111.2
C6A—C6—H6A	110.2	H15A—C15—H15B	109.1
C5—C6—H6A	110.2	N1—C16—C15	101.78 (11)
C6A—C6—H6B	110.2	N1—C16—H16A	111.4
C5—C6—H6B	110.2	C15—C16—H16A	111.4
H6A—C6—H6B	108.5	N1—C16—H16B	111.4
N12—C6A—C7	113.78 (9)	C15—C16—H16B	111.4
N12—C6A—C6	107.69 (9)	H16A—C16—H16B	109.3
C7—C6A—C6	114.12 (9)	O3—C17—O2	123.39 (11)
N12—C6A—H6C	106.9	O3—C17—C18	125.97 (12)
C7—C6A—H6C	106.9	O2—C17—C18	110.64 (11)
C6—C6A—H6C	106.9	C17—C18—H18A	109.5
O13—C7—O2	101.80 (8)	C17—C18—H18B	109.5
O13—C7—C6A	113.66 (9)	H18A—C18—H18B	109.5
O2—C7—C6A	105.80 (9)	C17—C18—H18C	109.5
O13—C7—C7A	103.59 (9)	H18A—C18—H18C	109.5
O2—C7—C7A	117.11 (9)	H18B—C18—H18C	109.5
C6A—C7—C7A	114.40 (9)	O5—C19—O4	122.84 (11)
C7—C7A—C8	101.53 (9)	O5—C19—C20	125.32 (12)
C7—C7A—C10A	104.21 (9)	O4—C19—C20	111.84 (11)
C8—C7A—C10A	100.39 (9)	C19—C20—H20A	109.5
C7—C7A—H7A	116.1	C19—C20—H20B	109.5
C8—C7A—H7A	116.1	H20A—C20—H20B	109.5
C10A—C7A—H7A	116.1	C19—C20—H20C	109.5
O4—C8—C9	111.63 (9)	H20A—C20—H20C	109.5
O4—C8—C7A	114.64 (10)	H20B—C20—H20C	109.5
C9—C8—C7A	93.72 (9)	O6—C21—O7	123.89 (11)
O4—C8—H8A	111.9	O6—C21—C10	127.16 (11)
C9—C8—H8A	111.9	O7—C21—C10	108.90 (10)
C7A—C8—H8A	111.9	O7—C22—H22A	109.5
O13—C9—C8	104.88 (9)	O7—C22—H22B	109.5
O13—C9—C10	104.91 (9)	H22A—C22—H22B	109.5
C8—C9—C10	100.21 (9)	O7—C22—H22C	109.5
O13—C9—H9A	115.1	H22A—C22—H22C	109.5
C8—C9—H9A	115.1	H22B—C22—H22C	109.5
C10—C9—H9A	115.1	H9B—O9—H9C	102.6
			
C12A—C1—C2—C3	−0.36 (19)	O13—C9—C10—C21	−58.89 (11)
C1—C2—C3—C4	−1.20 (19)	C8—C9—C10—C21	−167.43 (9)
C2—C3—C4—C4A	1.69 (19)	O13—C9—C10—C10A	66.63 (10)
C3—C4—C4A—C12A	−0.60 (19)	C8—C9—C10—C10A	−41.91 (10)
C3—C4—C4A—C5	175.67 (11)	C7—C7A—C10A—C11	56.86 (12)
C13—N1—C5—C4A	−139.61 (12)	C8—C7A—C10A—C11	161.69 (9)
C16—N1—C5—C4A	48.66 (15)	C7—C7A—C10A—C10	−72.93 (10)
C13—N1—C5—C6	92.85 (14)	C8—C7A—C10A—C10	31.90 (11)
C16—N1—C5—C6	−78.88 (14)	C21—C10—C10A—C11	−3.21 (15)
C4—C4A—C5—N1	54.92 (14)	C9—C10—C10A—C11	−121.72 (11)
C12A—C4A—C5—N1	−128.96 (12)	C21—C10—C10A—C7A	124.30 (11)
C4—C4A—C5—C6	−178.01 (11)	C9—C10—C10A—C7A	5.79 (11)
C12A—C4A—C5—C6	−1.89 (16)	C12A—N12—C11—O8	−6.56 (18)
N1—C5—C6—C6A	162.65 (10)	C6A—N12—C11—O8	164.24 (11)
C4A—C5—C6—C6A	36.59 (13)	C12A—N12—C11—C10A	168.47 (10)
C11—N12—C6A—C7	19.28 (15)	C6A—N12—C11—C10A	−20.73 (16)
C12A—N12—C6A—C7	−169.04 (9)	C7A—C10A—C11—O8	155.03 (11)
C11—N12—C6A—C6	−108.25 (12)	C10—C10A—C11—O8	−82.73 (14)
C12A—N12—C6A—C6	63.42 (12)	C7A—C10A—C11—N12	−20.19 (14)
C5—C6—C6A—N12	−67.46 (11)	C10—C10A—C11—N12	102.05 (12)
C5—C6—C6A—C7	165.21 (9)	C4—C4A—C12A—C1	−0.99 (17)
C9—O13—C7—O2	−114.71 (9)	C5—C4A—C12A—C1	−177.11 (11)
C9—O13—C7—C6A	132.01 (10)	C4—C4A—C12A—N12	171.70 (11)
C9—O13—C7—C7A	7.26 (11)	C5—C4A—C12A—N12	−4.43 (17)
C17—O2—C7—O13	164.37 (9)	C2—C1—C12A—C4A	1.46 (18)
C17—O2—C7—C6A	−76.61 (12)	C2—C1—C12A—N12	−171.09 (11)
C17—O2—C7—C7A	52.24 (14)	C11—N12—C12A—C4A	145.01 (11)
N12—C6A—C7—O13	−95.04 (11)	C6A—N12—C12A—C4A	−26.74 (15)
C6—C6A—C7—O13	29.09 (13)	C11—N12—C12A—C1	−42.35 (16)
N12—C6A—C7—O2	154.10 (9)	C6A—N12—C12A—C1	145.90 (11)
C6—C6A—C7—O2	−81.77 (11)	C5—N1—C13—O1	2.9 (2)
N12—C6A—C7—C7A	23.68 (13)	C16—N1—C13—O1	175.47 (12)
C6—C6A—C7—C7A	147.81 (10)	C5—N1—C13—C14	−176.14 (11)
O13—C7—C7A—C8	−37.81 (11)	C16—N1—C13—C14	−3.56 (15)
O2—C7—C7A—C8	73.31 (11)	O1—C13—C14—C15	163.23 (13)
C6A—C7—C7A—C8	−162.07 (9)	N1—C13—C14—C15	−17.77 (14)
O13—C7—C7A—C10A	66.17 (10)	C13—C14—C15—C16	30.76 (13)
O2—C7—C7A—C10A	177.28 (9)	C13—N1—C16—C15	23.13 (14)
C6A—C7—C7A—C10A	−58.10 (12)	C5—N1—C16—C15	−164.46 (11)
C19—O4—C8—C9	172.25 (10)	C14—C15—C16—N1	−32.34 (13)
C19—O4—C8—C7A	−82.75 (12)	C7—O2—C17—O3	−0.49 (17)
C7—C7A—C8—O4	−65.65 (12)	C7—O2—C17—C18	−179.47 (10)
C10A—C7A—C8—O4	−172.64 (9)	C8—O4—C19—O5	−4.77 (17)
C7—C7A—C8—C9	50.22 (10)	C8—O4—C19—C20	174.69 (10)
C10A—C7A—C8—C9	−56.77 (10)	C22—O7—C21—O6	−0.42 (18)
C7—O13—C9—C8	26.28 (11)	C22—O7—C21—C10	177.16 (10)
C7—O13—C9—C10	−78.82 (10)	C9—C10—C21—O6	99.42 (14)
O4—C8—C9—O13	71.45 (11)	C10A—C10—C21—O6	−14.78 (18)
C7A—C8—C9—O13	−46.92 (10)	C9—C10—C21—O7	−78.07 (12)
O4—C8—C9—C10	−179.98 (9)	C10A—C10—C21—O7	167.73 (10)
C7A—C8—C9—C10	61.65 (10)		

### Hydrogen-bond geometry (Å, °)

**Table:**

*D*—H···*A*	*D*—H	H···*A*	*D*···*A*	*D*—H···*A*
O9—H9B···O3^i^	0.89	1.98	2.8576 (12)	173
O9—H9C···O8	0.86	1.98	2.8365 (13)	177
O10—H10C···O1	0.91	2.06	2.9516 (15)	167

Symmetry codes: (i) *x*+1/2, *y*+1/2, *z*.
